# The effectiveness of proprioceptive training for improving motor function: a systematic review

**DOI:** 10.3389/fnhum.2014.01075

**Published:** 2015-01-28

**Authors:** Joshua E. Aman, Naveen Elangovan, I-Ling Yeh, Jürgen Konczak

**Affiliations:** ^1^Human Sensorimotor Control Laboratory, School of Kinesiology, University of MinnesotaMinneapolis, MN, USA; ^2^Center for Clinical Movement Science, University of MinnesotaMinneapolis, MN, USA

**Keywords:** balance, joint position sense, kinesthesia, proprioception, somatosensory, stroke, therapy

## Abstract

**Objective**: Numerous reports advocate that training of the proprioceptive sense is a viable behavioral therapy for improving impaired motor function. However, there is little agreement of what constitutes proprioceptive training and how effective it is. We therefore conducted a comprehensive, systematic review of the available literature in order to provide clarity to the notion of training the proprioceptive system.

**Methods**: Four major scientific databases were searched. The following criteria were subsequently applied: (1) A quantified pre- and post-treatment measure of proprioceptive function. (2) An intervention or training program believed to influence or enhance proprioceptive function. (3) Contained at least one form of treatment or outcome measure that is indicative of somatosensory function. From a total of 1284 articles, 51 studies fulfilled all criteria and were selected for further review.

**Results**: Overall, proprioceptive training resulted in an average improvement of 52% across all outcome measures. Applying muscle vibration above 30 Hz for longer durations (i.e., min vs. s) induced outcome improvements of up to 60%. Joint position and target reaching training consistently enhanced joint position sense (up to 109%) showing an average improvement of 48%. Cortical stroke was the most studied disease entity but no clear evidence indicated that proprioceptive training is differentially beneficial across the reported diseases.

**Conclusions**: There is converging evidence that proprioceptive training can yield meaningful improvements in somatosensory and sensorimotor function. However, there is a clear need for further work. Those forms of training utilizing both passive and active movements with and without visual feedback tended to be most beneficial. There is also initial evidence suggesting that proprioceptive training induces cortical reorganization, reinforcing the notion that proprioceptive training is a viable method for improving sensorimotor function.

## Introduction

Proprioceptive signals from mechanoreceptors of the joints, muscles, tendons, and skin are essential for the intact neural control of movement. The loss of proprioceptive afferents may affect the control of muscle tone, disrupts postural reflexes (Allum et al., 1998; Dietz, 2002; Rossignol et al., 2006) and severely impairs spatial (Gordon et al., 1995) as well as temporal aspects (Gentilucci et al., 1994) of volitional movement. Numerous neurological and orthopedic conditions are associated with proprioceptive and kinesthetic impairment such as stroke (Kenzie et al., 2014; Meyer et al., 2014), Parkinson's disease (PD) (Rickards and Cody, 1997; Khudados et al., 1999; Mongeon et al., 2009; Konczak et al., 2012), focal dystonia (Rosenkranz et al., 2000; Putzki et al., 2006), peripheral sensory neuropathies (Rothwell et al., 1982; Ghez et al., 1990), or injuries to ligaments, joint capsules, and muscles (Barrack et al., 1989; Lephart et al., 1994; Fridén et al., 1997).

Given the importance of proprioception for motor control, it has been argued that therapies aiming to restore motor function after injury should focus on training the proprioceptive sense. Numerous interventions claim to constitute a form of *proprioceptive training* that improves proprioception and aids motor recovery. Unfortunately, there is little agreement of what actually constitutes *proprioceptive training*, which may be partially owed to the fact that there are various definitions for the term *proprioception*. Broadly defined, proprioception refers to the conscious awareness of body and limbs and has several distinct properties: passive motion sense, active motion sense, limb position sense, and the sense of heaviness (Goldscheider, 1898). However, it has long been established that proprioception has an unconscious component in which proprioceptive signals are used for the reflexive control of muscle tone and the control of posture that has long been recognized (Sherrington, 1907). In order to distinguish between the conscious and unconscious processing of proprioceptive afferents it has been suggested to refer to kinesthesia as the conscious perception of limb and body position and motion and to reserve the term proprioception for referring to the unconscious processing of proprioceptive information (Konczak et al., 2009). However, this distinction is not without problems, because the term kinesthesia has also been used to indicate motion sense in distinction to position sense.

Recognizing that the processing of proprioceptive signals has conscious and unconscious components implies that the available methods for assessing proprioceptive function may only address one of the two aspects of proprioception. For assessing the perceptual aspect of proprioception, psychophysical thresholds represent the gold standard (Gescheider, 1985; Elangovan et al., 2014). In addition, determining a joint position error when matching the position of two homologous limbs (e.g., two arms), is the most easily acquired measure of proprioceptive function (Goble, 2010) and common in clinical practice. For determining the contribution of proprioceptive signals for balance control, many biomechanical measures have been employed such as latencies and amplitudes of electromyographic signals, joint kinematics or kinetics, or variables indicative of the postural sway of the body's center of mass. With respect to *proprioceptive training* this means that an intervention focusing on training the proprioceptive sense may train one or both aspects of proprioception, that is, the conscious perceptual or the unconscious or implicit sensorimotor aspect.

Further, it needs to be considered that proprioception is closely linked to movement. Unlike senses such as audition, where, for example, pitch perception can be trained in the absence of limb or body movement, proprioception requires movement. Thus, when evaluating the effectiveness of an intervention to improve proprioception, it may be difficult to isolate the sensory from a motor aspect of training. In fact, one can argue that any form of motor learning is associated with proprioceptive processing and thus may train proprioception. If one subscribes to such wide interpretation of *proprioceptive training*, the acquisition of motor skills, even those that are typically viewed to be visuomotor tasks such as reaching for objects or throwing darts, constitute a form of proprioceptive training. We would argue that such a wide definition of *proprioceptive training* is not helpful when addressing motor deficits that are known to be associated with proprioceptive dysfunction. Knowing that motor learning is inherently multisensory, it becomes impossible to discern if improvements in the acuity or sensitivity of one or more modality such as proprioception or vision contributed to improvements in motor performance, or whether changes in multisensory or sensorimotor integration are responsible. Consequently, in order to gain an understanding of the effectiveness of proprioceptive training, there ought to be a common understanding of what constitutes proprioceptive training. We therefore propose the following operational definition: *Proprioceptive training* is an intervention that targets the improvement of proprioceptive function. It focuses on the use of somatosensory signals such as proprioceptive or tactile afferents in the absence of information from other modalities such as vision. Its ultimate goal is to improve or restore sensorimotor function.

Because the term proprioceptive training has been widely used and claims of improved proprioception through specific interventions are commonly found in the literature, we applied the above definition to conduct a systematic review on the effectiveness of proprioceptive training. Specifically, we aimed to (a) document the array of outcome measures that have been used to assess proprioceptive training, (b) provide quantifiable data on the effectiveness of proprioceptive training intervention methods to improve somatosensory or sensorimotor performance, and (c) examine to what disease entities proprioceptive training has been applied. Finally, we critically discuss the main findings of this comprehensive review and provide recommendations for future research in this emerging field of study.

## Methods

### Literature search strategy and inclusion/exclusion criteria

A systematic search of the literature was performed using the databases of OVIDmedline, CINAHL, PsycINFO, and SCOPUS. A wide variety of keywords indicative of proprioception and proprioceptive training were used in the initial search. In combination with the terms *proprioception* and *training*, other search words included: rehabilitation, therapy, CNS, disease, nervous system, physical, therapy, therapeutic, exercise, training, effect, physiology, treatment, outcomes, movement, neuromuscular, facilitation, balance, functional, reaction time, biofeedback, behavior, combined modality, learning, perceptual, motor, processes, muscular, disorders, kinesthetic, kinesthetic, perception, discrimination, form, shape, human, and controlled. A full list of search terms can be found in Appendix A, Supplementary Material. The following inclusion criteria were employed: (1) A quantified pre- and post-treatment measure of proprioceptive function was reported (e.g., a psychophysical measurement *or* a clinical rating score). (2) An intervention or training program of any variable length or duration was applied believed to influence or enhance proprioceptive function. (3) Contained at least one form of treatment or outcome measure that relies on or is indicative of somatosensory function and is not confounded by information from other sensory modalities (e.g., visual feedback).

### Data extraction and reporting

The identified articles were then independently reviewed by three of the authors (Joshua E. Aman, Naveen Elangovan, I-Ling Yeh) and cross-checked to verify that each article met the above inclusion criteria. The following quantitative measures were obtained: First, the physiotherapy evidence database (PEDro) scale was applied in order to get a measure on study data validity and data interpretability (Verhagen et al., 1998). Scores were reported only for those studies that contained a comparable control group (See Table 1). Second, Cohen's *d* was calculated for each study where sufficient data were provided in order to quantify effect size, i.e., the standardized mean difference of an effect. In order to calculate Cohen's *d* for between groups (between two independent groups, a control group and an intervention group), the following formula was used:

(1) $$d_{s}=\frac{{\bar{X}}_{1}-\;{\bar{X}}_{2}}{\sqrt{\frac{\left(n_{1}-1\right)SD_{1}^{2}+(n_{2}-1)SD_{2}^{2}}{n_{1}+n_{2}-2}}}$$

where *d_s_* refers to the standardized mean difference between two groups of independent observations for the sample. The numerator is the difference between means of the two groups of observations and the denominator is the pooled standard deviation (Lakens, 2013). When calculating Cohen's *d* for correlated samples (those that utilized a pre/post-test without an independent control group), the following formula was used:

(2) $$d_{z}=\frac{M_{diff}}{\sqrt{\frac{\sum {\left(X_{diff}-M_{diff}\right)}^{2}}{N-1}}}$$

where *d_z_* refers to the standardized mean difference of effect size for within-subjects. The numerator is the difference between the mean (*M*) of the difference scores and the comparison value μ (in our case μ = 0). The denominator is the standard deviation of the difference scores (Lakens, 2013).

**Table 1:** **All reviewed studies categorized by intervention type**.

**Intervention type**	**References**	**Disease entity**	**Anatomical locations**	**Outcome measures**	**PEDro**
**ACTIVE MOVEMENT/BALANCE TRAINING**					
Balance training	Diracoglu et al., 2005	Osteoarthritis	Knee and whole body	JPS, WOMAC, SF-36, isokinetic knee extension strength, 10-m walking time, 10 stairs climbing time	6
	Hilberg et al., 2003	Hemophilia	Knee and whole body	Single leg stance, knee position sense	N/A^2^
	Risberg et al., 2007	ACL reconstruction	Knee and whole body	**Cincinnati Knee Score**, VAS-pain, VAS-global knee function, muscle strengthen, balance index	8
	Kerem et al., 2001	Cerebral palsy	Ankle and whole body	SEP, lower extremity ROM, modified Ashworth scale	4
	Eils and Rosenbaum, 2001	Ankle injury	Whole body	JPS, muscle reaction time, sway distance	3
	Eils et al., 2010	Ankle injury	Whole body	JPS, muscle reaction time, ankle injury incidence rates	3
	Kynsburg et al., 2006	Chronic lateral ankle instability	Whole body	Slope box test	N/A^2^
	Kynsburg et al., 2010	Healthy	Whole body	Slope box test	N/A^2^
	Panics et al., 2008	Healthy	Whole body	JPS	1
	Sekir and Gür, 2005	Osteoarthritis	Whole body	JPS, motion sense, Romberg's test, muscle strength	6
	Badke et al., 2011	Stroke	Whole body	**BBS**, TUG, DGI, SIS, ABC scale	N/A^1^
	Westlake and Culham, 2007	Geriatric	Whole body	**COP velocity**, FAB scale, ABC scale	6
Multi-joint active movement	Röijezon et al., 2009	Chronic neck pain	Neck	COP components, neck ROM, movement time, peak velocity, jerk index, variable error in neck JPS, VAS of pain, SF-36, NDI, TSK, Self-efficacy scale, DASH	N/A^1^
	de Oliveira et al., 2007	Stroke	Hand	FMA, modified Ashworth Scale, BI	N/A^1^
	Hocherman et al., 1988	Healthy	Arm	Reaching error	N/A^1^
	Hocherman, 1993	Healthy	Arm	Reaching error	N/A^1^
	Robin et al., 2004	Healthy	Arm	Reaching error	5
	Wong et al., 2012	Healthy	Arm	Arm tracking error, reaching velocity	6
	Casadio et al., 2009a	Stroke	Arm	Reaching kinematics	N/A^1^
	Jan et al., 2008	Osteoarthritis	Knee	JPS of knee, walking speed, FIS	7
	Lin et al., 2007	Osteoarthritis	Knee	JPS of knee, walking speed, WOMAC, strength of knee extensors and flexors	6
	Lin et al., 2009	Osteoarthritis	Knee	JPS of knee, walking speed, WOMAC	6
	Jacobson et al., 1997	Healthy	Arm and whole body	JPS of shoulder	N/A^2^
Single-joint passive vs. active movement	Beets et al., 2012	Healthy	Wrist	Movement error and variability	6
Multi-joint passive vs. active movement	Wong et al., 2011	Healthy	Arm	Movement speed and accuracy, discrimination the direction of passive movement	3
	Kaelin-Lang et al., 2005	Healthy	Thumb	Thumb acceleration, MEP	N/A^1^
**PASSIVE MOVEMENT TRAINING**					
Single-joint passive movement	Carel et al., 2000	Healthy	Wrist	Sensorimotor cortex activation	3
	Dechaumont-Palacin et al., 2008	Stroke	Wrist	Motor cortex activation, NIHSS, BI	3
	Ju et al., 2010	Healthy	Knee	JPS of knee	N/A^1^
**SOMATOSENSORY STIMULATION TRAINING**					
Electrical stimulation and rehabilitation therapy	Yozbatiran et al., 2006	Stroke	Wrist and finger	Motion sense, position sense, hand function test, hand movement scale	6
Magnetic stimulation	Struppler et al., 2003	Stroke	Finger	Modified Ashworth scale, finger kinematics	N/A^1^
Acupuncture	Liu et al., 2009	Stroke	Whole body	COP area	8
Thermal stimulation and movement training	Chen et al., 2011	Stroke	Whole body	FMA, BBS, PASS, Modified Motor Assessment Scale, FAC, modified Ashworth	7
				Scale	
Vibration	Rosenkranz et al., 2008	Focal dystonia	Hand	SICI (with behavioral proprioceptive training)	N/A^2^
	Rosenkranz et al., 2009	Focal dystonia	Hand	SICI, task-specific performance, self-assessment, BFM Scale, TCS	N/A^2^
	Chouza et al., 2011	Parkinson's disease	Whole body	TUG, Functional reach test	6
	Haas et al., 2006	Parkinson's disease	Whole body	Knee tracking error	N/A^2^
	van Nes et al., 2004	Stroke	Whole body	COP velocity, number of weight shifting	N/A^2^
Vibration with active movement training	Cordo et al., 2009	Stroke	Wrist or ankle	Muscle torque, ankle or wrist tracking error, gait analysis, SIS	N/A^1^
	Conrad et al., 2011	Stroke	Wrist	Movement smoothness, tracking errors	4
Vibration and balance training	Merkert et al., 2011	Stroke	Whole body	Tinetti gait test, BBS, BI, TUG, functional ability of the ééé lower back	4
Vibration and rehabilitation therapy	van Nes et al., 2006	Stroke	Whole body	**BBS**, TCT, Rivermead Mobility Index, BI, FAC, pressure sensitivity on hallux by monofilament	8
	Ebersbach et al., 2008	Parkinson's disease	Whole body	**Tinetti balance score, s**tand-walk-sit test, UPDRS, walking speed, pull test, posturography	4
**SOMATOSENSORY DISCRIMINATION TRAINING**					
	Carey and Matyas, 2005	Stroke	Wrist	WPST, TDT, FMT	N/A^1^
	Lynch et al., 2007	Stroke	Feet and ankle	BBS, DPT, Semmes-Weinstein monofilaments, ILAS	8
	Mace et al., 2008	Healthy	Wrist	SICI, ICF, MEP area	6
	Carey et al., 1993	Stroke	Hand	Texture discrimination test, proprioceptive discrimination test	N/A^1^
	Bakan and Thompson, 1967	Healthy	Hand	Point of subjective equality based on perceptual judgments	2
**COMBINED/MULTIPLE SYSTEM TRAINING**					
Multisensory stimulation and active movement training	Klages et al., 2011	Dementia	Whole body	**Sharpened Romberg test, Functional reach test, TUG**, frequency of falls	5
Multisensory stimulation and balance training	Missaoui and Thoumie, 2009	Sensory ataxia resulting from either ataxic neuropathy or multiple sclerosis	Whole body	BBS, Functional Reach Test, TUG, COP area	N/A^1^
Somatosensory discrimination and active movement training	McKenzie et al., 2009	Focal dystonia	Arm	Graphesthesia, Kinesthesia, Localization, Stereognosis, CAFÉ 40, upper limb ROM, Hand muscle strength	N/A^2^

*Abbreviations: ABC, Activities-specific Balance Confidence; FIS, Bandi Functional Incapacity Score; BI, Barthel Index; BBS, Berg Balance Scale; BFM, Burke-Fahn-Marsden Scale; COP, center of pressure; DASH, Disablity of Arm, Shoulder and Hand; DPT, Distal Proprioception Test; FAC, Functional Ambulation Classification; FMA, Fugl–Meyer Assessment; FMT, Fabric Matching Test; JPS, joint position sense; MEP, motor-evoked potential; MI, Motricity Index; MRC, Medical Research Council scale; NDI, Neck Disability Index; ICF, intra-cortical facilitation; ILAS, Iowa Level of Assistance Scale; ROM, range of motion; SEP, somatosensory evoked potential; SICI, short-interval intracortical inhibition; SIPT, Sensory Integration and Praxis Test; SIS, Stroke Impact Scale; TCS, Tubiana-Chamagne Scale; TCT, Trunk Control Test; TDT, Tactile Discrimination Test; TSK, TAMPA Scale of Kinesiophobia; UPDRS, Unified Parkinson's Disease Rating Scale' WOMAC, Western Ontario and McMaster Universities Osteoarthritis Index; WPST, Wrist Position Sense Test; N/A^1^, No control group in the study; N/A^2^, No comparable control group in the study (e.g., substantially less control subjects compared to testing subjects, no comparable intervention performed on control group). Bold outcome measures indicate primary outcome*.

Third, because within-subject pre/post-scores were the most consistently reported data among all included studies and because of the wide variety of applied intervention/training protocols and measurements, data were converted into a percentage of change from pre- to post-treatment in order to compare the effectiveness of training across a range of outcome measures and disease entities. For example, one study might require active hand movement in a visual tracking task while another passively moved a limb to a specified position. Or, some studies reported psychophysical thresholds while others used joint-position matching error or balance scores as a measure of proprioceptive function. The percent of improvement was calculated by dividing the difference between pre- and post-test by the pre-test measurement [(Posttest − Pretest)/Pretest]. For example, a post-treatment improvement of 3.9 points in Tinneti Gait Test with a pretest of 6.4 yields a 61% improvement ([10.3 – 6.4]/6.4).

## Results

### Initial search results and final included studies

**Figure 1:**
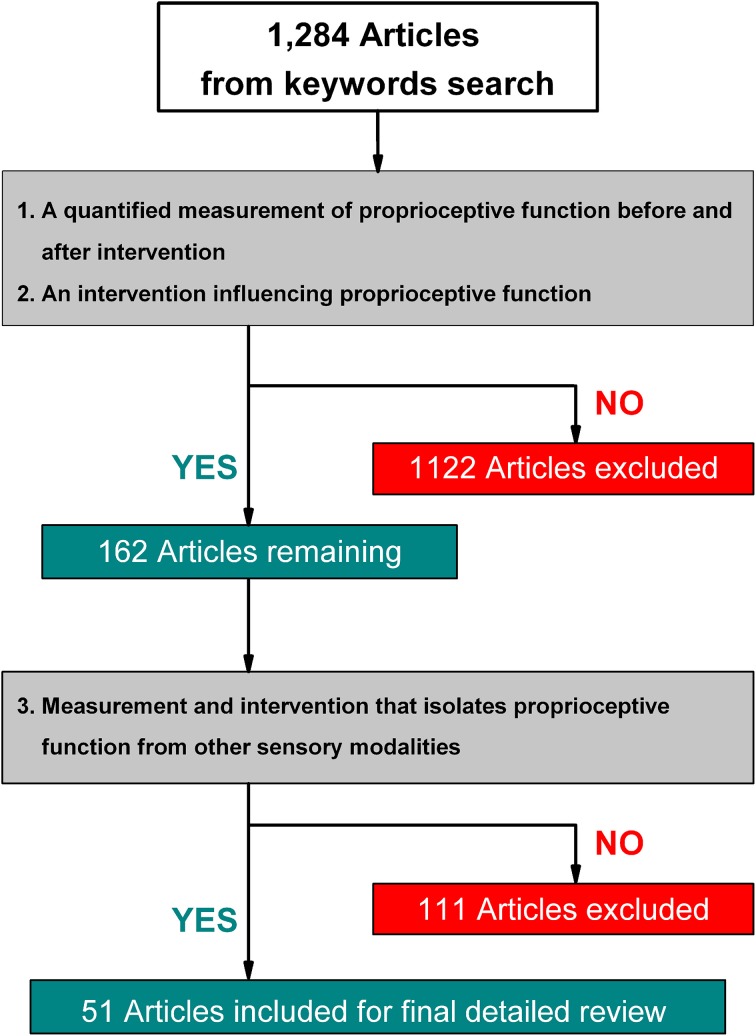
**Flow diagram of study selection process**.

The initial keyword search yielded 1284 articles. Three authors (Joshua E. Aman, Naveen Elangovan, I-Ling Yeh) reviewed all abstracts independently and applied criteria #1 and #2. After applying these criteria 162 articles remained. That is, the vast majority of articles (1122) did not report a quantifiable measure of proprioceptive function or did not include an intervention. Of those 162 articles, 111 studies were excluded, because they employed tasks where multimodal sensory information was always available during training or testing, and thus, the proprioceptive training aspect of the study was impossible to assess. We included those studies that utilized measurements indicative of proprioceptive function (e.g., clinical rating scales with subsections indicative of proprioceptive function). Typically, these studies involved measuring postural control where vision was available (see Figure 1 for an overview). The final 51 articles examined a total of 1854 subjects with sample sizes ranging from 5 to 186 participants. Study populations included healthy participants [20 articles], stroke [21], PD [4], focal dystonia [3], multiple sclerosis and ataxia neuropathy [2], cerebral palsy [1], dementia [1], osteoarthritis of the knee [7], chronic ankle instability [6], anterior cruciate ligament (ACL) reconstruction [2], chronic neck pain [2], and hemophilia [1]. Relative to the trained anatomical locations, articles focused on whole body/posture [22 articles], neck [1], shoulder/elbow [8], hand/wrist [16], knee [8], and ankle [3]. Note that some articles included more than one disease entity or anatomical location.

### Classification by outcome measures

**Figure 2:**
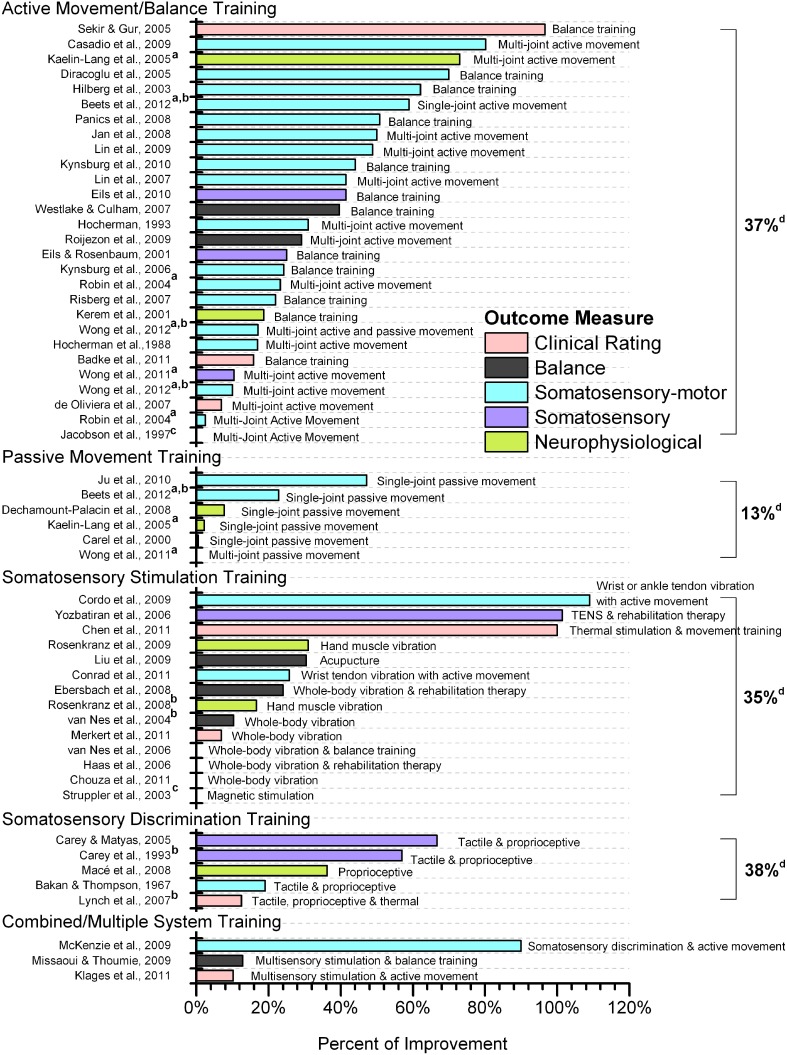
**Effectiveness of proprioceptive training by type of intervention**. Somatosensory, somatosensory-motor, balance and neurophysiological outcome measures were measured by device or instrument. If multiple outcome measures were reported from a single study that fell within the same classification (e.g., multiple clinical rating measures), only the most favorable result was reported. ^a^Studies providing two types of intervention. ^b^Values estimated from figures of the original article. ^c^Significant difference between pre- and post-test with no exact data reported. ^d^Mean percentage of improvement of each category. Studies not reporting exact data were not included in calculating the mean. Abbreviations: WBV, whole body vibration. TENS, transcutaneous electrical nerve stimulation.

In general, outcome measures were either based on a clinical rating scale or were obtained via some type of sensor involving a device (e.g., manipulandum for forearm joint position measurement, passive motion apparatus for deriving psychophysical thresholds, or force platform for measures of posture). In order to structure the wide variety of outcome measures, we assigned each study to one of five categories. All studies that exclusively used clinical rating scales as outcome measures were pooled into one category (*clinical rating*). The device dependent measurements were divided into four separate categories: *somatosensory*, *somatosensory-motor*, *balance*, and *neurophysiological*. A somatosensory measure consisted of thresholds or joint position errors obtained while the limb or body was moved passively, the somatosensory-motor category included articles reporting the same variables (thresholds, matching errors), but these outcomes were obtained when at least one limb or body segment was actively moved by the subject. The balance category included measures of whole body sway such as sway area, or displacement of the center of pressure (COP). The neurophysiological category included studies that reported neurophysiological measures associated with somatosensory or proprioceptive processing such as somatosensory evoked potentials (SEPs), or measures from functional MRI or transcranial magnetic stimulation (TMS). To get a sense of the heterogeneity of the reported outcome measures, we here provide a brief synopsis below. In addition, Figure 2 lists the outcome measure category of each article.

#### Clinical rating

Twelve studies (Sekir and Gür, 2005; van Nes et al., 2006; de Oliveira et al., 2007; Lynch et al., 2007; Ebersbach et al., 2008; Missaoui and Thoumie, 2009; Badke et al., 2011; Chen et al., 2011; Chouza et al., 2011; Klages et al., 2011; Merkert et al., 2011) utilized clinical rating scales as an outcome measure. Nine different clinical scales were used including balance scales (e.g., Berg Balance Scale, PASS), gait scales (e.g., Tinneti Gait score), a trunk control test, and impairment severity scales (e.g., FMA, Motor Assessment Scale). Other forms of clinical scales used were the NIH stroke scale, Barthel index and the Western Ontario and McMaster Universities Arthritis Index.

#### Balance

Ten studies utilized devices to measure balance which included force platform posturography and other COP-related measurements (Jacobson et al., 1997; Eils and Rosenbaum, 2001; van Nes et al., 2004; Risberg et al., 2007; Westlake and Culham, 2007; Ebersbach et al., 2008; Liu et al., 2009; Missaoui and Thoumie, 2009; Röijezon et al., 2009; Eils et al., 2010).

#### Somatosensory

Eleven articles reported an outcome measure of somatosensory function, which included thresholds or joint position matching errors during passive movement position or tactile discrimination tests (Carey et al., 1993; Eils and Rosenbaum, 2001; Carey and Matyas, 2005; Sekir and Gür, 2005; van Nes et al., 2006; Yozbatiran et al., 2006; Risberg et al., 2007; Eils et al., 2010; Ju et al., 2010; Wong et al., 2011).

#### Somatosensory-motor

Twenty-five studies fell into this category. They involved at least one form of active movement such as single or multi-joint position matching, tracking or reaching tasks, or haptic discrimination tests. Typical reported variables were joint position matching errors, psychophysical thresholds, spatial reaching error, and other limb kinematic measures such as movement time, velocity, or range of motion (Bakan and Thompson, 1967; Hocherman et al., 1988; Hocherman, 1993; Jacobson et al., 1997; Hilberg et al., 2003; Struppler et al., 2003; Robin et al., 2004; Diracoglu et al., 2005; Sekir and Gür, 2005; Haas et al., 2006; Kynsburg et al., 2006, 2010; Lin et al., 2007, 2009; Jan et al., 2008; Panics et al., 2008; Casadio et al., 2009a; Cordo et al., 2009; McKenzie et al., 2009; Röijezon et al., 2009; Ju et al., 2010; Conrad et al., 2011; Wong et al., 2011, 2012; Beets et al., 2012).

#### Neurophysiological

Seven studies provided a neurophysiological correlate of proprioceptive processing based on reporting somatosensory or motor evoked potentials (MEPs) or measures of intracortical inhibition and cortical activation (Carel et al., 2000; Kerem et al., 2001; Kaelin-Lang et al., 2005; Dechaumont-Palacin et al., 2008; Mace et al., 2008; Rosenkranz et al., 2008, 2009).

### Effectiveness of proprioceptive training by type of intervention

Researchers commonly used the term proprioceptive training to either indicate some form of intervention that aimed to improve the accuracy of the proprioceptive system (e.g., measurement outcomes that assessed proprioceptive acuity or sensitivity) or to indicate a proprioception-based intervention (interventions based solely on proprioceptive information). We established five main classifications of training to categorize the approach of the reviewed studies: *active movement/balance training*, *passive movement training, somatosensory stimulation training, somatosensory discrimination training*, *combined/multiple system training*. Table 1 lists all studies grouped according to these five categories. It includes the studied disease entity, anatomical location of the applied procedure or intervention, outcome measures and PEDro score. Table 2 lists all studies for which Cohen's *d* was calculated for within-group comparisons categorized by intervention type. Table 3 provides a list of those studies for which Cohen's *d* was calculated for between-group comparisons categorized by intervention type. Finally, Figure 2 provides an overview (percent change from pre- to post-test) of the degree of sensory or motor improvement resulting from the various intervention methods. For the sake of simplicity we refer to all training or therapeutic regimens as proprioceptive training. However, one needs to recognize that a given study might have included more than one component of intervention.

**Table 2:** **Cohen's *d* measures for between-group comparisons categorized by intervention type**.

**Study**	**Disease entity**	**Outcome measure**	**Primary/secondary measure**	**Sample size**		**SD_pooled_**	**Cohen's *d***	**95% CI**	
				**Tx**	**Control**			**LL**	**UL**
**ACTIVE MOVEMENT/BALANCE TRAINING**									
Eils and Rosenbaum, 2001	Ankle injury	Somatosensory: passive JPS	–	20	10	0.435	0.000^*^	−0.793^*^	0.793^*^
		Balance: COP max-ML	–	20	10	3.275	0.092^*^	−0.668^*^	0.850^*^
		Balance: COP max-AP	–	20	10	6.756	−0.089^*^	−0.847^*^	0.672^*^
		Balance: COP SD-ML	–	20	10	0.849	0.118^*^	−0.643^*^	0.876^*^
		Balance: COP SD-AP	–	20	10	1.533	−0.130^*^	−0.889^*^	0.643^*^
		Balance: COP total path	–	20	10	95.471	−0.341^*^	−1.102^*^	0.426^*^
Eils et al., 2010	Ankle injury	Somatosensory: passive JPS	–	91	81	0.756	0.926^*^	0.609^*^	1.240^*^
		Balance: COP max-ML	–	91	81	3.694	0.217^*^	−0.084^*^	0.517^*^
		Balance: COP max-AP	–	91	81	4.295	0.629^*^	−0.321^*^	0.934^*^
		Balance: COP SD-ML	–	91	81	0.953	0.210^*^	−0.091^*^	0.510^*^
		Balance: COP SD-AP	–	91	81	1.168	0.685^*^	0.376^*^	0.992^*^
		Balance: COP total path	–	91	81	72.941	−0.315^*^	−0.616^*^	−0.014^*^
Kerem et al., 2001	Cerebral palsy	Neurophysiological: SEPs from the stimulation on the right limb	–	10	14	9.295	0.245^*^	−0.572^*^	1.057^*^
		Neurophysiological: SEPs from the stimulation on the left limb	–	10	14	9.159	0.321^*^	−0.500^*^	1.134^*^
Lin et al., 2009	Osteoarthritis	Somatosensoy-motor: active JPS error	–	36	36	2.001	1.299^*^	−0.610^*^	0.823^*^
		Somatosensoy-motor: active JPS error	–	36	36	1.404	1.710^*^	−0.393^*^	1.047^*^
Panics et al., 2008	Healthy	Somatosensoy-motor: active JPS error of dominant leg	–	20	19	2.219	1.388^*^	0.678^*^	2.083^*^
		Somatosensoy-motor: active JPS error of non-dominant leg	–	20	19	2.244	0.931^*^	0.263^*^	1.588^*^
Risberg et al., 2007	ACL reconstruction	Somatosensory: TTDPM	Secondary	31	34	0.657	−0.030^*^	−0.517^*^	0.456^*^
		Somatosensoy-motor: static balance index	Secondary	31	34	176.487	0.085	−0.402	0.571
		Somatosensoy-motor: dynamic balance index	Secondary	31	34	320.701	0.461	−0.034	0.953
Wong et al., 2011	Healthy	Somatosensory: uncertainty area in passive movement direction detection	–	25	25	2.800	0.525^*^	−0.042^*^	1.087^*^
	Geriatric	Balance: COP velocity	Primary	17	19	0.987	0.699^*^	0.02^*^	1.370^*^
Westlake and Culham, 2007		Balance: COP velocity with secondary task	Primary	17	19	0.735	0.802^*^	0.116^*^	1.478^*^
**SOMATOSENSORY STIMULATION TRAINING**									
Ebersbach et al., 2008	Parkinson's disease	Clinical: Tinetti gait score	Primary	10	11	2.178	0.597	−0.287	1.466
		Balance: linear tilting distance of tilting board	Secondary	10	11	544.042	1.746^*^	0.711^*^	2.748^*^
Liu et al., 2009	Stroke	Somatosensoy-motor: COP area with eyes open	–	15	15	37.239	0.107^*^	−0.610^*^	0.823^*^
		Somatosensoy-motor: COP area with eyes closed	–	15	15	39.613	0.331^*^	−0.393^*^	1.047^*^
Merkert et al., 2011	Stroke	Clinical: BBS	–	25	23	9.627	0.322^**^	−0.250^**^	0.890^**^
		Clinical: Tinetti gait score	–	11	8	2.842	0.493^**^	−0.440^**^	1.411^**^
van Nes et al., 2006	Stroke	Clinical: BBS	Primary	27	26	13.556	−0.037	0.575	−0.502
**COMBINED/MULTIPLE SYSTEM TRAINING**									
Klages et al., 2011	Dementia	Clinical: Sharpened Romberg test	Primary	9	10	7.402	0.770^*^	−0.177^*^	1.696^*^

*Twelve studies reported sufficient information for computing Cohen's d value. Among the remaining studies, 15 studies did not include a control group, 6 studies included non-equivalent groups and 16 studies did not report sufficient information. Kynsburg et al. (2010) was excluded because the control group only performed the baseline assessment. Yozbatiran et al. (2006) was excluded because the outcome measure were nominal*.

* *Direction of effect size was converted that increment indicates improvement*.

** *The dependent variable is the difference between pre- and post-score. Abbreviations: ACL, anterior cruciate ligament; AP, anterior-posterior; BBS, Berg Balance Scale; CI, confidence interval; COP, center of pressure; JPS, joint position sense; LL, lower limit; ML, medial-lateral; SD, standard deviation; SEP, somatosensory evoked potential; TTDPM, threshold to detection of passive movement; Tx, Treatment group; UL, upper limit*.

**Table 3:** **Cohen's *d* measures for within-group comparisons categorized by intervention type**.

**Study**	**Disease entity**	**Outcome measure**	**Primary/secondary measure**	**Sample size**	**Cohen's *d***	**95% CI**	
						**LL**	**UL**
**ACTIVE MOVEMENT/BALANCE TRAINING**							
Badke et al., 2011	Stroke	Clinical: BBS	Primary	29	1.295	0.723	1.858
Lin et al., 2009	Osteoarthritis	Somatosensory-motor: active JPS reposition error	–	36	1.450^*^	U/A	U/A
**SOMATOSENSORY STIMULATION TRAINING**							
Merkert et al., 2011	Stroke	Clinical: BBS	–	25	1.140	0.631	1.636
		Clinical: Tinetti gait score	–	11	1.300	0.322	1.381
**COMBINED/MULTIPLE SYSTEM TRAINING**							
Missaoui and Thoumie, 2009	Sensory ataxia resulting from either ataxic neuropathy or multiple sclerosis	Clinical: BBS	–	24	0.877	0.400	1.340
		Somatosensory-motor: COP area with eyes open	–	24	0.129^*^	0.274^*^	0.53^*^
		Somatosensory-motor: COP area with eyes closed	–	24	0.026^*^	−0.310^*^	0.426^*^
		Somatosensory-motor: COP area with standing on the foam	–	24	0.434^*^	0.011^*^	0.781^*^
Risberg et al., 2007	ACL reconstruction	Somatosensory-motor: static balance index	–	24	0.52	U/A	U/A
		Somatosensory-motor: dynamic balance index	–	24	0.600	U/A	U/A

*Five studies had either provided sufficient information for computing Cohen's d or reported it in the publication. U/A: Unable to apply calculation of CI because of insufficient information. Forty three studies did not provide sufficient information for computing Cohen's d. Three studies were not suitable for computing Cohen's d (Carey et al., 1993 and Carey and Matyas, 2005: single-case design; Yozbatiran et al., 2006: nominal outcome measures)*.

* *Direction of effect size was converted that increment indicates improvement. Abbreviations: BBS, Berg Balance Scale; CI, confidence interval; COP, center of pressure; LL, lower limit; JPS, joint position sense; U/A, unable to apply; UL, upper limit*.

With respect to the calculation of Cohen's *d*, no relationship became apparent between intervention type and the calculated magnitude of effect, which is consistent with the observed heterogeneity of outcomes across all reviewed studies. Of the 51 reviewed articles 42 articles provided sufficient data to calculate an effect size. Thirty-two articles were considered as between-group effects (range of effect size: −0.037–1.746) (see Table 2) and 10 articles were considered as within-group effects (range of effect size: 0.026–1.45) (see Table 3). According to Cohen (1992), the magnitude of effect can be categorized into small (>0.2), medium (>0.5), and large (>0.8) effects. In total, 13 articles showed insufficient effect sizes (less than 0.2) with 7 of those articles showing effect sizes of 0 or less, 9 articles showed small effect sizes, 8 articles showed medium effect sizes, and 12 articles showed large effects sizes. It is noteworthy that those interventions employing a form of active joint position sense training most often resulted in large effect sizes (>0.8) (see Tables 2, 3).

#### Active movement/balance training

The active movement/balance training included studies in which participants *actively* moved a limb, limb segment or the whole body. Approximately half of the studies [26 out of 51 articles] fell into this category. The following interventions were employed: single-joint active movements [1 article], single-joint passive and active movements [1], multi-joint passive and active movements [1], multi-joint active movements [12], and whole body balance training [12].

Multi-joint active movements consisted of upper limb tasks such as reaching to or grasping a target with or without additional sensory feedback (e.g., vision) (Hocherman et al., 1988; Hocherman, 1993; Robin et al., 2004; de Oliveira et al., 2007; Casadio et al., 2009b; Wong et al., 2012) or lower limb tasks such as stepping on specific targets (Lin et al., 2007, 2009; Jan et al., 2008). In general, studies applying multi-joint, active movement training tasks reported improvements that ranged widely from 2.5 to 80.2% (mean: 39%) from pre- to post-test (see Figure 2).

With respect to goal-directed reaching or grasping with or without assistance (Hocherman et al., 1988; Hocherman, 1993; Robin et al., 2004; de Oliveira et al., 2007; Casadio et al., 2009b; Wong et al., 2011, 2012), robot-aided upper limb movements guided by online haptic feedback (assist-as-needed force) showed the greatest degree of accuracy improvement (Casadio et al., 2009b). After 10-h of training, nine chronic stroke patients (vision occluded) substantially reduced reaching endpoint error by 81.4% (mean change: 53.2–9.9 cm) and by 80% when visual feedback was present. It is interesting to note that the presence of vision did not result in superior post-test performance. In comparison, when healthy subjects trained with both visual and proprioceptive information available, endpoint error was reduced by approximately 24% (from 20.5 to 15.5 mm) after 720 trials (Robin et al., 2004). When the same healthy subjects could only rely on proprioceptive information and knowledge of results, the improvement was less pronounced compared to receiving both visual and proprioceptive feedback, showing approximately a 2% reduction in endpoint error from pre- to post-test (Robin et al., 2004). Providing auditory feedback as an error signal reduced endpoint error in healthy subjects by 17–31% after 75–130 trials (Hocherman et al., 1988; Hocherman, 1993). This implies that both visual and auditory feedback yield approximately the same degree of improvement when trying to improve reaching accuracy. Equivalent goal-directed actions with the lower limb such as stepping on specific targets while visual error feedback was provided reduced knee reposition error in patients with osteoarthritis by 42–48% (Lin et al., 2007, 2009; Jan et al., 2008).

Studies seeking to train balance applied a multitude of activities including walking and stair-stepping exercises, single and double leg balance exercises with and without vision, sit-to-stand exercises, standing, walking, or jumping on stable and unstable surfaces and sport specific exercises (Eils and Rosenbaum, 2001; Hilberg et al., 2003; Diracoglu et al., 2005; Sekir and Gür, 2005; Kynsburg et al., 2006, 2010; Risberg et al., 2007; Westlake and Culham, 2007; Panics et al., 2008; Eils et al., 2010; Badke et al., 2011). Across all studies pre- to post-test improvements ranged between 16 and 97% (mean: 41%). Three of these studies conducted active dynamic exercise training and examined knee position sense using active/passive joint matching tests (Diracoglu et al., 2005; Sekir and Gür, 2005; Panics et al., 2008) reporting reductions in knee joint position error between 15 and 63% as a result of training.

One study obtained additional knee joint position detection thresholds from patients with knee osteoarthritis (Sekir and Gür, 2005). Subjects with knee osteoarthritis started at a predetermined knee joint angle. Subsequently, the knee was moved at 1°s^−1^ toward either flexion or extension and subjects verbally indicated at what position they perceived movement and in which direction. After completing the 6-week training regimen, subjects were again tested and results showed their average detection thresholds for joint motion improved in both flexion (baseline: 2.3°, post-treatment: 1.3°) and extension (baseline: 2.4°, post-treatment: 1.5°). In a separate, passive-active knee joint position matching test they determined knee position sense acuity for three different reference positions (20, 45, and 70° joint angle). Based on a knee joint position error, improvement gains were computed ranging between 37 and 60% (Pre/post-test error for 20° reference: 8.8°/5.5°; 45° reference: 7.4°/3.0°; 70° reference: 6.7°/3.7°). In a subsequent active-active matching test 29–46% improvements were obtained (Pre/post-test error for 20° reference: 10.1°/4.4°; 45° reference: 6.8°/3.7°; 70° reference: 6.8°/4.5°).

In a study with chronic neck pain patients, participants manipulated a ball within an apparatus placed on their head, requiring them to control the ball by neck movements. They received only knowledge of results (i.e., whether they hit the target) without vision of the ball during training. Curiously, results showed an *increase* in neck repositioning error after training from 2.54 to 3.03 cm, a decrement of 19% that was accompanied by a 29% reduction in postural sway (mean sway area pre-test: 1.61 cm^2^; post-test: 1.41 cm^2^) (Röijezon et al., 2009).

With respect to training dosage it is noteworthy that most included balance training regimens lasted 6 weeks or more. Interestingly, the training by Risberg et al. (2007) consisting of balance exercises, dynamic joint stability exercises, plyometric exercises, agility drills, and sport-specific exercises lasted between 3 and 5 weeks and did not yield significant improvements in proprioceptive detection thresholds.

#### Passive movement training

**Figure 3:**
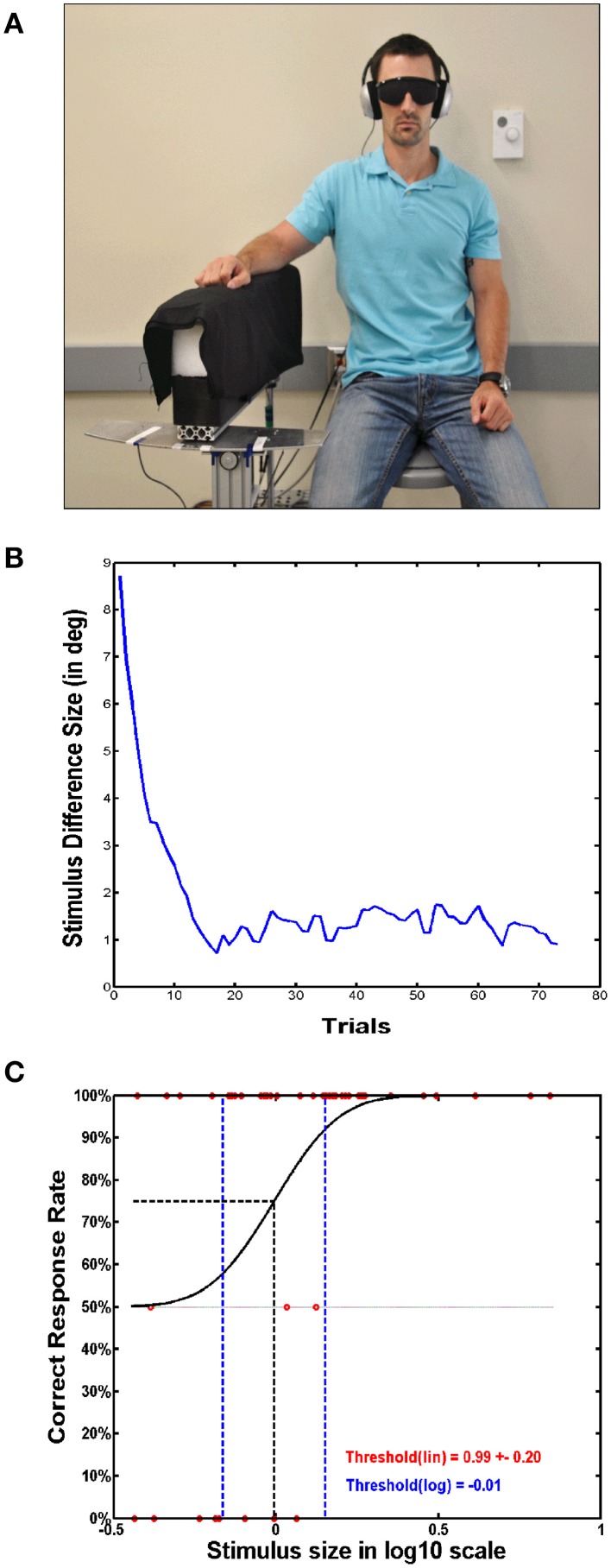
**Passive motion apparatus used for determining proprioceptive acuity and sensitivity**. **(A)** A subject sitting with their right arm resting on a passive motion apparatus (PMA). The PMA is used for passively moving the subject's arm, in this case specifically the elbow, in order to determine proprioceptive acuity and sensitivity. **(B)** Stimuli intensities are plotted across the trials performed. In this case, an adaptive algorithm can be used, which determines the next delivered stimulus based on the correctness of the subject's previous response. **(C)** A psychophysical function is deduced from the responses of the subject, with a correct response level of 75% taken as the just-noticeable-different threshold.

These interventions typically required some type of passive motion apparatus and focused either on single-joint (wrist or knee) (Carel et al., 2000; Dechaumont-Palacin et al., 2008; Ju et al., 2010; Beets et al., 2012) or multi-joint movement (thumb movement or assisted reaching via robotic arm) (Kaelin-Lang et al., 2005; Wong et al., 2011). Although there are several forms of apparatus' that have been used that are often customized for a specific joint or movement type, Figure 3 provides an example of such devices. Like active training protocols widely divergent rates of functional or sensory improvement were reported ranging from a 0 to 47% change from pre- to post-test. Most occluded vision of the moving limb (Carel et al., 2000; Dechaumont-Palacin et al., 2008; Wong et al., 2011; Beets et al., 2012) but some allowed vision (Wong et al., 2011; Beets et al., 2012) or provided additional synchronized auditory feedback (Dechaumont-Palacin et al., 2008). All studies except Ju et al. (2010) reported a range of 0–23% improvements.

Ju et al. (2010) reported the highest gains after passive movement training. They trained healthy subjects who received 60 repetitions of passive knee movement (10° to 100° range with average angular velocity of 120°/s). Visual feedback of knee movement was available, while an air splint wrapped around the knee blocked tactile information during passive movements. They showed a reduction of active-active knee joint matching error from 1.93° to 1.02° (47% improvement) and a reduction of active-passive matching error from 1.94 to 1.22 (37% improvement).

Three studies compared training with passive versus active movements (Kaelin-Lang et al., 2005; Wong et al., 2011; Beets et al., 2012). All three showed active movement training was more beneficial than passive movement in somatosensory-motor measures (e.g., endpoint error of a reaching task) and neurophysiological measures (MEP of the targeted thumb muscle) (Wong et al., 2011; Beets et al., 2012).

#### Somatosensory stimulation training

This type of training included various forms of stimulation that was geared exclusively to somatosensation. Ten studies applied muscle or vibro-tactile vibration ranging from whole-body vibration to local vibration of a single segment (van Nes et al., 2004, 2006; Haas et al., 2006; Ebersbach et al., 2008; Rosenkranz et al., 2008, 2009; Cordo et al., 2009; Chouza et al., 2011; Conrad et al., 2011). Other forms of stimulation included thermal stimulation (Chen et al., 2011), multi-somatosensory stimulation (Kaelin-Lang et al., 2005), magnetic stimulation (Struppler et al., 2003), electrical stimulation (Yozbatiran et al., 2006), and acupuncture (Liu et al., 2009).

Studies utilizing whole-body vibration during training mostly targeted patients with PD (Haas et al., 2006; Ebersbach et al., 2008; Chouza et al., 2011) or stroke (van Nes et al., 2004, 2006; Merkert et al., 2011). Typical vibration frequencies were either between 25 and 30 Hz or <10 Hz. Most studies applied vibration for a duration of less than 1 min per trial except Ebersbach and colleagues who used 15-min sessions of continuous vibration (Ebersbach et al., 2008). After four 45-s bouts of 30 Hz vibration with eyes closed, a significant reduction in COP displacement was observed in stroke patients while standing. When vibration was stopped and patients were allowed to open their eyes, COP velocity was reduced in a visually-guided weight-shift task (van Nes et al., 2004).

A randomized-clinical control study combined balance training simultaneously with whole-body vibration (15 sessions; 15–90 s of 35 Hz vibration per session) in geriatric stroke patients in addition to conventional geriatric rehabilitation therapy (Merkert et al., 2011). The balance exercises, including bridging in supine position or maintain sitting or standing balance, were carried out with subject's feet on the vibratory device. The 3-week combined training lead to a 61% mean improvement in the Tinetti Gait test score from pre- to post-test (6.4 points to 10.3 points) while the control group, who had only received conventional rehabilitation therapy, improved to a lesser extent (8.9–10.4 pts.).

Chouza et al. (2011) evaluated the acute effects of vibration training at frequencies of 3, 6, and 9 Hz compared to a placebo. They found a lack of significant differences in timed-up-and-go and functional reach before and after training. One possible reason for this null-effect is that vibration at these low frequencies does not induce sufficient activity in the Ia afferent fibers of muscle spindles to stimulate proprioceptive processing. A study that applied relatively higher vibration frequencies (60–70 Hz) actually yielded the highest degree of functional improvement (Cordo et al., 2009). Stroke patients alternated between a passively flexed and extended ankle joint while a vibration was alternately applied to lengthening muscles during the repeated flexion and extension movements (i.e., vibration was applied to the flexor muscles during extension and to the extensor muscles during flexion). This training task continued over a period of 6 months. Proprioceptive sense was tested by having subjects follow a ramp-and-hold position task in which they were to track a cursor visualized on a screen with a second cursor that indicated the angle of their ankle joint position. Training resulted in a 109% improvement in tracking accuracy. Based on the limited evidence provided by these studies it can be stated that vibration frequencies exceeding 30 Hz have shown to be most effective in functional outcomes (van Nes et al., 2006; Cordo et al., 2009; Conrad et al., 2011; Merkert et al., 2011).

#### Somatosensory discrimination training

This training focused on the ability to discriminate between two somatosensory stimuli. These discrimination training tasks consisted of haptic discrimination (e.g., active exploration of objects with the hand) (Bakan and Thompson, 1967), tactile discrimination (of textures) (Carey et al., 1993; Carey and Matyas, 2005; Lynch et al., 2007), wrist or ankle joint position discrimination (Carey et al., 1993; Carey and Matyas, 2005; Lynch et al., 2007), and wrist joint velocity discrimination tasks (Mace et al., 2008). Improvements in haptic or proprioceptive acuity ranged from 12 to 67% (mean improvement of 38%). The two studies training wrist joint position discrimination (with graded difficulty) as a training regimen for stroke patients showed improvements of 57–67% in wrist joint angle position matching. In absolute terms joint position error was reduced from approximately 25–30° at pre-test to 8–10° at post-test (Carey et al., 1993; Carey and Matyas, 2005). One of the shortcomings of these two studies was a small sample size (7 participants in total), which limits the generalization of the results. This caveat is warranted, because another study utilizing a similar approach of joint position discrimination to the ankle and/or toe joint(s) failed to show significant differences in the acuity of the joint position sense after 5 h of training. The measurement of proprioception used was the Distal Proprioception Test (DPT), which was the correctness of discrimination of toe positions (Lynch et al., 2007). One possible confounding factor could be that DPT is measured with a scale of less precision compared to joint position sense measurements using a device, which may then fail to detect possible changes. The other difference could be the shorter total treatment time (5 h) (Lynch et al., 2007) compared to approximately 7.5 to 10 h (Carey and Matyas, 2005).

#### Combined/multiple system training

Three studies used either multiple components of the three main categories mentioned above or utilized multi-sensory approaches. Noteworthy was a study by McKenzie et al. (2009) who combined both active movement training with somatosensory discrimination tasks to treat focal hand dystonia patients. Subjects with writers' cramp showed a 90% improvement in finger target location error (baseline error: 14 mm, post-intervention: 1.4 mm) and subjects with musician's cramp showed a 22% improvement (baseline error: 3.87 mm, post-intervention: 3 mm).

In summary, a wide variety of training regimes have been applied to improve proprioceptive and/or motor function. When reviewing the effectiveness data in Figure 2 it becomes evident that no particular training regimen is clearly superior to others. A general conclusion is that proprioceptive training can be very effective. Active movement, balance exercises, somatosensory stimulation and somatosensory discrimination training all yielded mean improvement rates of over 30%. In contrast, passive movement training was overall less successful. However, this assessment warrants a caveat, because the reported improvements show large within-training method variability. Part of this variability is likely owed to the fact that these training methods were applied to a number of divergent disease entities. We therefore provide below a second viewpoint that assesses the effectiveness of training for each clinical population.

### Effectiveness of proprioceptive training by type of disease entity

Proprioceptive training has been applied to healthy and a range of clinical populations mainly with neurological or orthopedic conditions. One aim of this review was to investigate, if such training has a differential effect on these populations. In other words, is there evidence indicating that proprioceptive training is especially beneficial in treating a specific type of disease. Appendix B in Supplementary Material provides an overview of the reviewed studies sorted according to disease entity. We broadly categorized the studies according to study sample: *healthy* adults, *neurological* patients, patients with *musculoskeletal disorders* and others.

#### Healthy adults

Studies on healthy adults help to assess to what extent proprioceptive or associated motor functions can be improved in the absence of disease. This is important for the evaluation of the effectiveness of specialized proprioceptive training for clinical populations. That is, data from studies on healthy subjects can serve as a baseline against which therapeutic success can be assessed. Based on the 14 reviewed studies that conducted proprioceptive training with healthy adults, a mean improvement rate of 26% was computed. However, outcome variability was high (range: 0–73%) and four studies reported no or very small gains (<5%).

#### Stroke

Cortical stroke has been the most investigated disease. Mean improvement across the 16 reviewed studies was 42%. In general, studies that employed a proprioceptive training regimen on any segment in the upper limb for multiple sessions demonstrated improvements in their outcome measures as compared to pre-intervention levels. Overall, somatosensory stimulation training was the training regimen that yielded the highest levels of improvement. For example, a 6-month vibration training for chronic stroke patients yielded a 109% improvement in accuracy in tracking task (Cordo et al., 2009).

#### Parkinson's disease/dystonia

Only three studies investigated patients with PD (because of the small sample we did not compute a mean improvement score). Whole body vibration was the only form of proprioceptive training studied in PD. Ebersbach et al. (2008) examined postural control of two groups of PD patients, one group receiving whole-body vibration as a training regimen and another group receiving conventional balance training exercises (e.g., balancing on a tilt board). To measure postural control, subjects stood on a tilt board and displacement of the board was taken as the measure of performance. After a 3-week (two 15-min sessions a day, 5 days a week) intervention the results showed a 33% reduction in displacement of the tilt board in the whole-body vibration group and a 23% *increase* in displacement of the tilt board in the balance exercise group. That is, in this study vibration facilitated balance training and enhanced the effectiveness of conventional balance training. In contrast, a study by Haas and colleagues of whole-body-vibration training (5 sessions of 60 s each) of subjects with PD produced no significantly different results in balance performance when compared to conventional physical therapy (Haas et al., 2006). One reason for the differential results between the two previously stated studies may be in how they measured performance; Ebersbach et al. (2008) measured errors in oscillating target reproduction via knee extension/flexion; Haas et al. (2006) utilized the Tinetti Balance scale and posturography to measure treatment effects. Keeping in mind that studies evaluating PD often show relatively high variability in results, another possible reason for difference in results between the two stated studies may be in the treatment times used during their intervention. Haas et al. (2006) provided subjects a total of 5 min of whole-body vibration while subjects in the study by Ebersbach et al. (2008) received a total of 450 min of whole-body vibration.

Focal dystonia is a disease known to be associated with proprioceptive impairment that is likely linked to their motor symptoms (Konczak and Abbruzzese, 2013). There is initial evidence that proprioceptive training improves the proprioceptive acuity in patients with focal hand dystonia (Rosenkranz et al., 2008, 2009). In a comprehensive study of patients with musician's dystonia and writer's cramp, subjects participated in an 8-week therapy regimen that initially focused on somatosensory retraining (McKenzie et al., 2009). Progressive motor retraining was subsequently introduced and progressed. Each group demonstrated significant improvement with sensorimotor retraining as measured by localization, graphesthesia, and kinesthesia tests. Motor control improved particularly in participants with writer's cramp. After therapy, their mean target reaching error had decreased tenfold from 14 mm (±17.51 mm) to 1.4 mm (±0.59 mm).

#### Musculoskeletal disease

Musculoskeletal conditions included chronic neck pain, ACL reconstruction, ankle injury, and osteoarthritis. Typical training regimens consisted of active multi-joint or whole body movement as well as whole body balance training (Eils and Rosenbaum, 2001; Diracoglu et al., 2005) and treatment durations ranged from 1 day (Lin et al., 2007) to 6 weeks (Kynsburg et al., 2010). Among the musculoskeletal conditions, proprioceptive training proved most beneficial for improving function in knee osteoarthritis (mean improvement rate: 61%; see Appendix B in Supplementary Material). With respect to knee osteoarthritis, the high effectiveness of proprioceptive training is underlined by the fact that the minimum functional improvement was 42% (Lin et al., 2007).

In summary, therapeutic success through proprioceptive training was achieved in a variety of neurological and orthopedic diseases. Based on the data provided in Tables 2, 3 and Appendix B in Supplementary Material, it is apparent that proprioceptive training can be beneficial for rehabilitation of neurological based injury such as stroke, PD and dystonia, and also for musculoskeletal conditions such ACL reconstruction, ankle instability and osteoarthritis. However, this conclusion warrants a caveat. Given the current lack of carefully randomized, controlled studies with sufficient sample sizes and given the wide range of the reported effect sizes of proprioceptive training, one needs to be careful not to generalize to the extent that all reported interventions are effective.

### The role of non-proprioceptive feedback during proprioceptive training

Several studies used non-proprioceptive feedback in order to improve proprioceptive function. Hocherman et al. (1988) demonstrated that active proprioceptive training in the form of target reaching assisted with acoustic feedback reduces target reaching error immediately after training. However, when the subjects had to reach to remembered targets from the prior training session (approximately 2 days prior), the efficiency of reaching reduced by approximately 25%. Further evaluation showed that this reduction in target reaching efficiency occurs mainly due to the inaccurate internal representation of the space rather than inaccurate motor planning. This conclusion was based on the training of one hand to reach proprioceptive targets and testing the other hand for accuracy in reach position (Hocherman, 1993). Further, other studies used passive or active movement training to show how the presence of feedback might affect sensorimotor function. When no feedback was given, there were no significant differences of corticospinal excitability before and after passive wrist movement (Mace et al., 2008), or between passive and active training groups (Beets et al., 2012). With visual feedback, active training was shown to be superior to passive training. A significant improvement in spatial accuracy of an active wrist tracking test (with feedback) was shown following training with an active tracking task versus a group performing passive wrist tracking that included online visual feedback and fixed auditory feedback (Beets et al., 2012). Thus, active training in the presence of visual feedback showed significant improvements in proprioceptive acuity in healthy subjects.

In summary, the results of the above studies suggest that for improving proprioceptive function, a training regimen including an active movement component is superior to interventions that only employ passive limb motion.

### Neural correlates of proprioceptive training

A subset of seven studies documented neural changes associated with improved sensorimotor function after proprioceptive training. Despite this small sample, the studies highlight that measurable neural changes occur as a function of proprioceptive-based training. For example, Kerem et al. (2001) measured SEPs in patients with cerebral palsy after 3 months of Bobath's neurodevelopmental therapy in conjunction with the use of Johnstone pressure splints. When their SEPs were compared to patients completing therapy without the use of splints, the splinted patients showed a 21% and 17% decrease in posterior tibial nerve peak vertex latency of their right and left leg SEPs. Changes in MEPs as a function of proprioceptive training have been reported in healthy subjects. After 30 min of passive flexion and extension of the thumb, MEP amplitudes decreased by 19% and 26% in agonist and antagonist musculature, respectively. After half an hour of active thumb flexion/extension training, MEP amplitudes increased in the agonist by 19% and decreased by 29% in the antagonist musculature (Kaelin-Lang et al., 2005). Conversely, Mace and colleagues showed a 45% increase in MEP amplitude of the hand/wrist musculature following 1 h of passive flexion and extension of the wrist. Increased excitability was shown up to 1 h post-treatment (Mace et al., 2008).

Rosenkranz and colleagues had documented that healthy non-musicians show a characteristic pattern of the sensorimotor organization of the hand motor cortex, with reduced intracortical inhibition in projections to the vibrated muscle and increased intracortical inhibition in “surrounding” projections to the non-vibrated ones. This pattern was less well differentiated in healthy musicians and lost in musician's dystonia (Rosenkranz et al., 2008). In a follow-up study (Rosenkranz et al., 2009), musicians with task-specific focal dystonia received proprioceptive training that lasted for 15 min and involved repeated cycles of muscle vibration (2 s on, 2 s off) applied in equal amounts to several hand muscles. The proprioceptive input due to vibration restored sensorimotor organization, as measured by short-interval intracortical inhibition, in pianists with musician's dystonia to the pattern seen in healthy pianists. Crucially, task-specific motor control also improved.

Two studies used functional magnetic resonance imaging (fMRI) to evaluate cortical activity associated with proprioceptive training (Carel et al., 2000; Dechaumont-Palacin et al., 2008). Carel and colleagues showed increased activation in the primary sensorimotor cortex and supplementary motor area (SMA) following passive flexion/extension wrist movements (20 min/day – 5 days/week – 4 weeks) in healthy subjects. Results also showed a redistribution of activity within the SMA, indicating functional reorganization (Carel et al., 2000). A study by Dechamount-Palacin and colleagues showed increased ipsilesional and decreased contralesional activation in stroke patients following 4 weeks of a standard Bobath rehabilitation program. However, a second group of patients completed 4 weeks of a standard Bobath rehabilitation program along with passive wrist movement training. In this second group of patients, imaging data showed increased contralesional activation in the SMA, prefrontal cortex, inferior parietal cortex, secondary sensory cortex, and ventral premotor cortex. The investigators concluded that the involvement of the SMA may be important for the recovery of proprioceptive function after stroke (Dechaumont-Palacin et al., 2008).

In summary, there is increasing evidence that proprioceptive training is associated with reorganization within the sensorimotor cortex and SMA. Currently, the complete neural network that leads to cortical reorganization and ultimately to improved proprioceptive function has not been fully identified. However, the known proprioceptive dysfunction after basal ganglia lesions (Konczak et al., 2009) and the vast connectivity of the basal ganglia-thalamus-cortical loop (Lenglet et al., 2012) make it quite plausible that this functional loop contributes to proprioceptive-motor learning.

## Discussion

This systematic review on proprioceptive training aimed to provide comprehensive metadata on (a) the type of outcome measures used to assess proprioceptive training, (b) the effectiveness of specific proprioceptive training interventions for improving somatosensory or sensorimotor performance, and (c) the disease entities that had been selected for proprioceptive training.

Evaluating scientific evidence on the effectiveness of proprioceptive training has been challenging given the heterogeneity in training methods and obtained measurements. While the keyword guided search yielded 1284 prospective articles, our initial analysis revealed that the majority of publications used terms such as proprioceptive training or sensory retraining rather loosely. Yet, even the 51 studies that fulfilled all inclusion criteria (see Figure 1) and were subjected to detailed review constituted a heterogeneous sample due to differences in methodology and measurements. Many studies used a variety of active and passive movement procedures, often combining both methods for their analysis, which makes it difficult to assess the contribution of each training method for improving proprioceptive and/or motor function. Another factor that very likely contributed to the variability of the reported effectiveness was the widely divergent duration of the intervention ranging from a single session to 6 months. Despite these challenges, our approach of reporting training-related improvement rates offered a way to assess effectiveness of the procedures across the wide range of applied training methods and reported outcome measures. Based on the analysis of improvement rates the following general conclusions can be made:

First, proprioceptive training can be effective in improving proprioceptive function. The majority of studies (29 out of 51) reported improvement rates above 20%. This assessment is corroborated when considering effect size calculations. Twenty out of the 42 studies for which effect size could be calculated showed medium to large effect sizes (<0.5). There is initial evidence that training methods of progressive rehabilitation that includes some form of somatosensory stimulation, utilizing a combination of both passive and active movement with and without exteroceptive feedback, are most beneficial (typically reporting large effect sizes of >0.8; see Tables 2, 3). Second, longer lasting interventions seem to produce greater benefits. Training regimens lasting 6 weeks or longer tended to yield relatively higher improvements in proprioceptive and/or motor function (Eils and Rosenbaum, 2001; Diracoglu et al., 2005; Sekir and Gür, 2005; Kynsburg et al., 2006, 2010; Lin et al., 2007, 2009; Jan et al., 2008; Panics et al., 2008; Cordo et al., 2009; Eils et al., 2010), although somatosensory stimulation has shown to yield very rapid gain within a single session or few hours of intervention (Rosenkranz et al., 2008; Conrad et al., 2011). Third, proprioceptive training is applicable to a wide range of clinical populations. Patients suffering from proprioceptive impairment may benefit from the procedure disregarding if the cause of the impairment is neurological or musculoskeletal in nature.
However, the reader needs to recognize that the above conclusions warrant a caveat and are preliminary in nature. While the reported effectiveness data are, in many respects, impressive, there are also reports that showed no or only minimal functional gains. The reasons for this discrepancy are not necessarily obvious. However, small differences in protocol may yield large differences in outcome. Thus, in our opinion, there is a clear need for developing standardized protocols based on the available preliminary evidence. Ideally, those protocols can then be tested by several investigators independently. In this review, employing the PEDro rating scale showed that 20 of the 51 articles did not use a comparable control group during testing and those that did showed a wide range of PEDro scores (1–8, maximum of 10). Further, there is very little clarity on what constitutes an optimal training dosage. That is, what are best practices for session duration, number of weekly sessions, and overall duration of the intervention? While a 6-week period has shown to produce positive results, it is by no means clear that this is an optimal duration. Related to dosage is the notion of retention. Only a few studies investigated retention of function over a period of 45 min (van Nes et al., 2004) or 6 weeks (van Nes et al., 2006). Thus, for many of the applied training methods we have no firm data on the associated decay of learning, which would be important knowledge to have for any follow-up therapy. Another aspect rarely addressed so far concerns the specificity or generalizability of training. While the majority of studies reported gains in proprioceptive and/or motor performance, surprisingly little is known, if such gains transfer beyond the trained tasks. The data documenting cortical reorganization following treatment are helpful (Carel et al., 2000; Dechaumont-Palacin et al., 2008; Fiehler and Rosler, 2010), because they hint that proprioceptive training induces generic neural changes that are not task-specific. However, there is still no comprehensive evidence available to support this claim across the reviewed training methods.
Finally, terminological clarity on how *proprioceptive training* is defined is dearly needed to overcome the current epistemological quagmire. During the first-pass screening of all articles that were produced by the keyword search, it became apparent that many studies use this term loosely, often referring to tasks that involve multimodal sensorimotor learning. As we have argued at the beginning of this review, if one subscribes to the view that any form of sensorimotor learning constitutes proprioceptive training, then the term carries very little meaning scientifically and for the clinical community. If, on the other hand, one considers proprioceptive training to be a form of unimodal sensory training or a form of specialized sensorimotor learning, then this should be explicitly stated to avoid terminological confusion and vagueness. This has been recognized in the past by several researchers who offered the opinion to avoid the term proprioceptive training when referring to training forms that may show motor improvements but fail to document associated improvements in proprioceptive function (Ashton-Miller et al., 2001; Haas et al., 2006). We therefore would advocate that there is a need to define the term proprioceptive training and we here offer the following operational definition: *Proprioceptive training* is an intervention that targets the improvement of proprioceptive function, focusing on the use of somatosensory signals such as proprioceptive or tactile afferents in the absence of information from other modalities such as vision. Its ultimate goal is to improve or restore sensory and/or sensorimotor function. Although, applying such a definition implies increased scientific effort by demanding the provision of direct measures of proprioceptive function (such as psychophysical thresholds), we would contend that its application has value in guiding future research seeking to exploit the proprioceptive sense to improve a wide range of motor function.

In summary, our aim was to review the available literature in order to provide clarity to the notion of training the proprioceptive system. There is converging evidence that proprioceptive training can yield meaningful improvements in somatosensory and sensorimotor function. However, there is a clear need for further work. With respect to improving motor function, an amalgamated approach may be most advantageous. Those forms of training incorporating both passive and active movements (i.e., proprioceptive and sensorimotor information) with and without visual feedback appear to be most beneficial. There is also initial evidence suggesting that proprioceptive training induces cortical reorganization, providing evidence for the notion that proprioceptive training is a viable method for improving motor function.

### Conflict of interest statement

The authors declare that the research was conducted in the absence of any commercial or financial relationships that could be construed as a potential conflict of interest.
