# Supplementary material for: The effectiveness of proprioceptive training for improving motor function: a systematic review
Source: Front Hum Neurosci. 2015 Jan 28;8:1075. doi: 10.3389/fnhum.2014.01075 (PMC4309156; doi:10.3389/fnhum.2014.01075)
Supplement: Supplementary file 1 [file DataSheet1.PDF]

Appendix A

Search Terms

Proprioception, rehabilitation, therapy, CNS disease, central nervous system diseases, proprioceptive training, physical therapy, therapeutic exercise, training effect, treatment outcomes, movement, neuromuscular facilitation, balance training, physical, functional status, reaction time, outcomes, biofeedback, behavior therapy, combined modality therapy, perceptual motor learning, motor processes, muscular disorders, kinaesthetic perception, discrimination, form and shape perception, learning, humans, controlled study, kinesthetic, disease, nervous system, therapeutic, exercise, physiology, outcomes, balance, perceptual, motor behavior, detection
